# EGaIn-based thermal management for high heat flux electronics

**DOI:** 10.1016/j.isci.2026.117822

**Published:** 2026-10-15

**Authors:** Jin Young Joung, Hyeonjong Na, So Hee Yoon, Yong-hee Jeong, Moohyun Lee, Carmel Majidi, Han Kim

**Affiliations:** 1 Department of Mechanical Engineering, Tech University of Korea, Siheung-si 15073, Republic of Korea; 2 Department of Mechanical Engineering, Carnegie Mellon University, Pittsburgh, PA 15213, USA

**Keywords:** eutectic gallium-indium, EGaIn, liquid metal, thermal management, thermal interface materials, high heat flux electronics

## Abstract

The increasing power density of modern electronic systems makes efficient thermal management essential for maintaining device performance, reliability, and operational lifetime. Among emerging thermal management materials, eutectic gallium-indium (EGaIn) has attracted attention because it uniquely combines metallic thermal conductivity with room-temperature fluidity, enabling efficient heat transfer and conformal contact with interfaces. In this review, we examine EGaIn as an adaptive thermal management platform and connect its material properties with the thermal functions they enable, their implementation in cooling systems, and the associated engineering challenges. We discuss how EGaIn enhances heat dissipation through interfacial heat transfer, liquid metal cooling, and thermally conductive composites, and review applications in batteries, high-performance electronics, electronic packaging, and flexible devices. We discuss challenges including oxidation, leakage, corrosion, electrical insulation, and long-term reliability, together with engineering strategies to address them. Finally, we provide perspectives for developing reliable EGaIn-based thermal management technologies for next-generation electronic and energy systems.

## Graphical abstract

**Figure:**
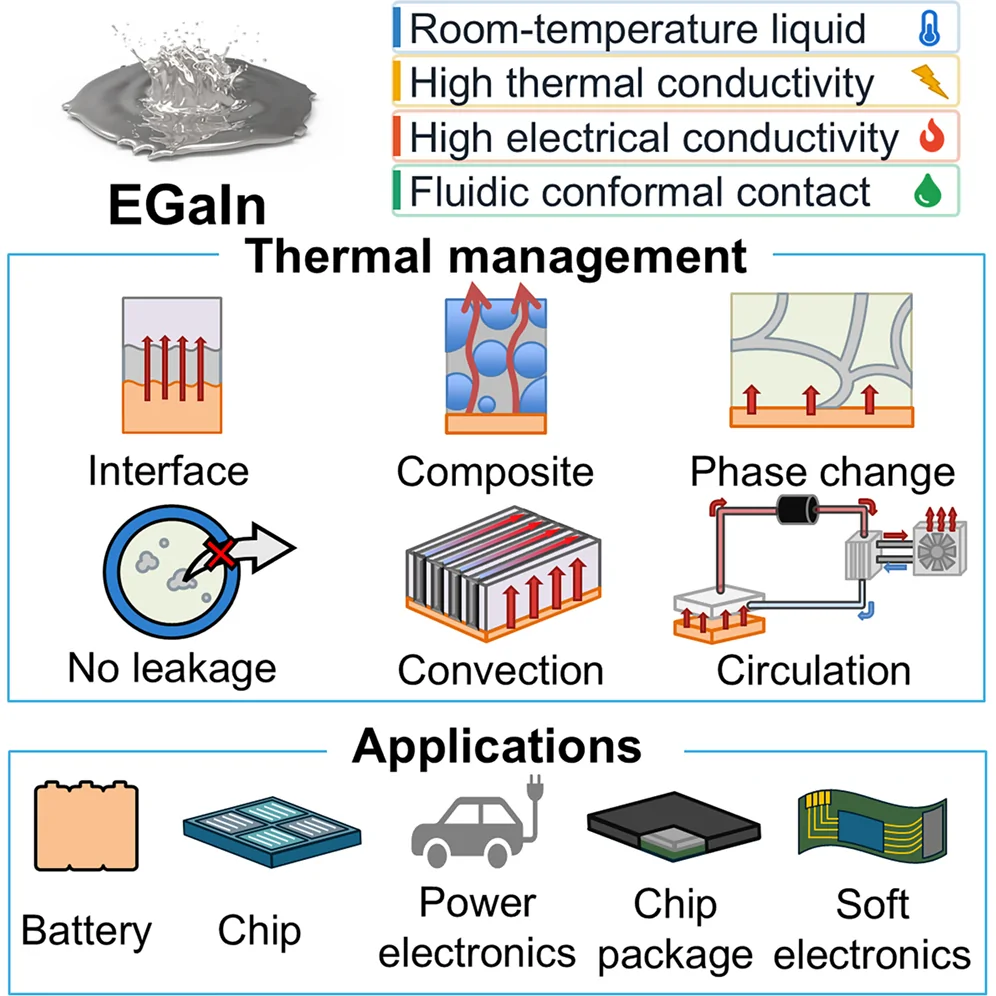

## Teaser

Energy systems; Thermal property; Devices

## Introduction

Thermal management has emerged as one of the most critical challenges in modern electronic systems.^1^ Device performance is no longer governed solely by electrical design, as semiconductor scaling has resulted in increasing power densities and highly localized heat generation.^2^^,^^3^ As processor nodes shrink and device integration increases, heat flux becomes concentrated within progressively smaller regions, promoting the formation of localized hotspots that are difficult to dissipate using conventional cooling technologies.^4^ Therefore, thermal management has become a key determinant of device performance, reliability, and operational lifetime. Despite substantial differences in structure and functionality, technologies such as high-performance processors,^5^ artificial intelligence (AI) accelerators,^6^ power devices,^7^ high-brightness light-emitting diodes (LEDs),^8^ and battery modules^9^ share a common challenge of efficiently transferring heat away from regions where it is generated. The rapid development of flexible and wearable bioelectronics^10^^,^^11^^,^^12^^,^^13^ and self-powered energy-harvesting^14^^,^^15^^,^^16^^,^^17^ devices has further increased the demand for efficient thermal management. This issue becomes particularly severe in densely integrated architectures such as chip arrays, where heat generation is highly localized.^18^ In such systems, local heat fluxes can exceed tens of W/cm^2^, and inadequate heat removal rapidly results in temperature non-uniformity and the formation of persistent hotspots.^18^^,^^19^

The consequences of excessive heat accumulation extend beyond reduced thermal efficiency.^19^ Elevated temperatures adversely affect the electrical characteristics of semiconductor devices and accelerate the accumulation of thermomechanical stress, which reduces long-term reliability and service life.^20^ For example, in insulated-gate bipolar transistor (IGBT) and silicon carbide metal-oxide-semiconductor field-effect transistor (SiC MOSFET) modules, mitigation of load-induced junction-temperature fluctuations has been shown to reduce accumulated damage by approximately 77%, leading to nearly a 4-fold increase in projected device lifetime.^21^ These observations highlight that device performance, durability, and safety are strongly influenced by both heat generation and heat dissipation efficiency and reliability. Therefore, thermal management should be considered a design parameter of equal importance to electrical design.^22^

Heat generated within electronic systems is dissipated through a thermal pathway comprising the semiconductor device,^23^ package,^24^ thermal interface material (TIM),^25^ heat spreader,^26^ heat sink,^27^ and cooling medium.^28^ A major thermal bottleneck often occurs at interfaces, where microscopic surface roughness creates air-filled voids that reduce the effective contact area and impede heat transfer.^29^ TIMs mitigate this resistance by filling these gaps, with their performance governed primarily by thermal conductivity, bond-line thickness (BLT), and contact resistance.^5^^,^^30^ Conventional TIMs exhibit an inherent trade-off between thermal conductivity and conformability. Thermal greases provide excellent surface conformity but relatively low thermal conductivity and may undergo pump-out during thermal cycling,^31^^,^^32^ whereas highly filled polymer pads become increasingly stiff.^33^^,^^34^ Solders and metallic foils provide substantially higher thermal conductivity but offer limited conformability and may introduce thermomechanical reliability concerns.^35^^,^^36^^,^^37^ Consequently, simultaneously achieving high thermal conductivity, low contact resistance, mechanical compliance, and long-term stability remains a central challenge in TIM design.^5^

Gallium (Ga)-based liquid metals have attracted considerable attention as a TIM because they offer a promising solution to the long-standing trade-off of thermal conductivity and mechanical reliability.^38^^,^^39^^,^^40^ Unlike conventional TIMs, liquid metals combine metallic thermal transport characteristics with liquid-like deformability.^38^ Their fluid nature enables intimate contact with rough and irregular surfaces, while their metallic bonding provides thermal conductivities substantially greater than those of conventional polymer-based materials.^41^ Liquid metals can simultaneously reduce interfacial thermal resistance and improve heat-transfer efficiency.^5^ For example, Ga-based liquid metals used as chip-level TIMs have been shown to lower both interfacial thermal resistance and device operating temperatures compared with commercial thermal greases.^5^ Furthermore, liquid metal composites containing tungsten microparticles have achieved thermal conductivities exceeding 60 W/m·K while maintaining long-term stability.^42^ Other studies have reported self-solidifying liquid metal composites that are initially applied as conformable liquids and subsequently solidify through Ga-Cu intermetallic formation, which suppresses pump-out and enhances thermal-cycling reliability.^43^^,^^44^ These developments demonstrate that liquid metals uniquely integrate efficient metallic heat-transfer pathways and excellent interfacial adaptability within a single material platform.^35^

Among Ga-based liquid metals, eutectic gallium-indium (EGaIn) is one of the most extensively investigated systems.^45^^,^^46^^,^^47^ EGaIn remains liquid near room temperature without requiring external heating, and the formation of a thin native gallium oxide (Ga_2_O_3_) layer enables unique rheological behavior.^48^ The oxide skin allows EGaIn to maintain structural integrity below a critical stress while permitting flow when sufficient force is applied.^45^ These characteristics offer significant advantages for thermal management applications. However, they are accompanied by important engineering challenges. The liquid nature of EGaIn introduces leakage risks, while its high electrical conductivity can lead to short circuits and device failure when unintended electrical contact occurs.^36^ Furthermore, Ga reacts with engineering metals such as copper (Cu) and aluminum (Al), resulting in corrosion, intermetallic formation, and liquid metal embrittlement.^49^^,^^50^ In practice, successful implementation depends on diffusion barriers, protective coatings, or carefully engineered structures to mitigate material degradation.^40^ Recent reviews have broadly covered Ga-based liquid metals, including their fundamental chemistry, composites, phase-change media, and use as heat-transfer fluids.^51^^,^^52^^,^^53^^,^^54^^,^^55^

In this review, we focus specifically on EGaIn containing 75.5 wt % Ga and 24.5 wt % In as the reference material and distinguish EGaIn evidence from results obtained with Ga, GaInSn, Ga_68.5_In_21.5_Sn_10_ (Galinstan), and other Ga-based alloys. EGaIn studies are prioritized, while studies of other alloys are included only when they establish a transferable mechanism or cooling architecture and are clearly identified by composition. By linking EGaIn properties with interfacial transport, cooling strategy selection, application constraints, and reliability challenges, this review provides an engineering framework for evaluating EGaIn thermal management under high heat flux conditions.

## Material properties of EGaIn for thermal management

Liquid metals, including pure Ga, Ga-based alloys, and other low-melting-point (LMP) metallic systems, have attracted considerable interest for thermal management applications because they combine high thermal conductivity with fluidity and mechanical compliance.^18^^,^^54^^,^^56^ Among the available liquid metal candidates, EGaIn has been widely studied because it combines near-room-temperature fluidity with metallic thermal conductivity.^45^ Pure Ga has favorable thermal characteristics, but its melting point near room temperature can result in solidification during storage or operation.^57^ Table 1 compares pure Ga, EGaIn, Ga_68.5_In_21.5_Sn_10_, and water under the stated property conditions. Galinstan has an equilibrium melting temperature of 284 K and should be treated as related alloy rather than EGaIn.^58^^,^^62^ Its representative room temperature thermal conductivity is 25 W/m·K, although reported values vary with composition, oxide content, and measurement method.^42^^,^^58^ The equilibrium melting temperature is listed separately from lower, history-dependent solidification temperatures that can arise from supercooling. In contrast, EGaIn possesses a eutectic temperature of 289 K, significantly lower than that of pure Ga (303 K), enabling liquid-state operation under typical indoor thermal-management conditions while requiring freeze protection below its equilibrium melting point.^45^

**Table 1:** Comparison of the physical and thermophysical properties of pure gallium (Ga), eutectic gallium-indium (EGaIn), a Ga-based alloy, and water

Property	Pure Ga	EGaIn	Ga-based alloy	Water
Composition (wt %)	Ga_100_	Ga_75.5_In_24.5_	Ga_68.5_In_21.5_Sn_10_	H_2_O
Melting point (K)	303	289	283–254 (at supercooled)	273
Density (kg/m^3^)	6,090	6,280	6,360	997
Thermal conductivity (W/m·K)	29.3	25.9–26.4	25	0.610
Specific heat (J/kg·K)	370	350	365	4,180
Electrical conductivity (S/m)	3.7 × 10^6^	3.3–3.5 × 10^6^	3.3 × 10^6^	5.5 × 10^−6^
Surface tension (N/m)	0.71	≈0.62	0.54–0.59	0.072
Thermal resistivity (m·K/W)	0.034	0.038	0.040–0.043	1.6
Dynamic viscosity (mPa·s)	≈2.0	≈1.99	1.89–2.40	0.891
Ref.	Handschuh-Wang et al.,^58^ Yao et al.,^59^ and Assael et al.^60^	Handschuh-Wang et al.^58^ and Plevachuk et al.^61^	Handschuh-Wang et al.,^58^ Liu et al.,^62^ and Plevachuk et al.^63^	Brini et al.^64^

EGaIn denotes an alloy containing 75.5 wt % Ga and 24.5 wt % In. Unless otherwise indicated, the listed values are representative literature values at or near room temperature. Water properties are reported at 298 K and 101.3 kPa, except for its melting point. Thermophysical properties may vary with temperature, composition, surface oxidation, and measurement method.

The attractiveness of EGaIn for thermal management originates from its unique combination of room-temperature fluidity, high thermal and electrical conductivity, low eutectic temperature, and conformal contact (Figure 1).^5^^,^^8^^,^^31^^,^^40^ These characteristics facilitate the formation of efficient heat-transfer pathways while minimizing interfacial thermal resistance and mitigating localized hotspot formation. Rather than functioning solely as a thermally conductive material, EGaIn serves as an adaptive thermal medium capable of maintaining intimate interfacial contact under high-heat-flux conditions. Therefore, understanding how these intrinsic properties contribute to thermal management performance is essential for the rational design of EGaIn-based cooling systems.

**Figure 1:**
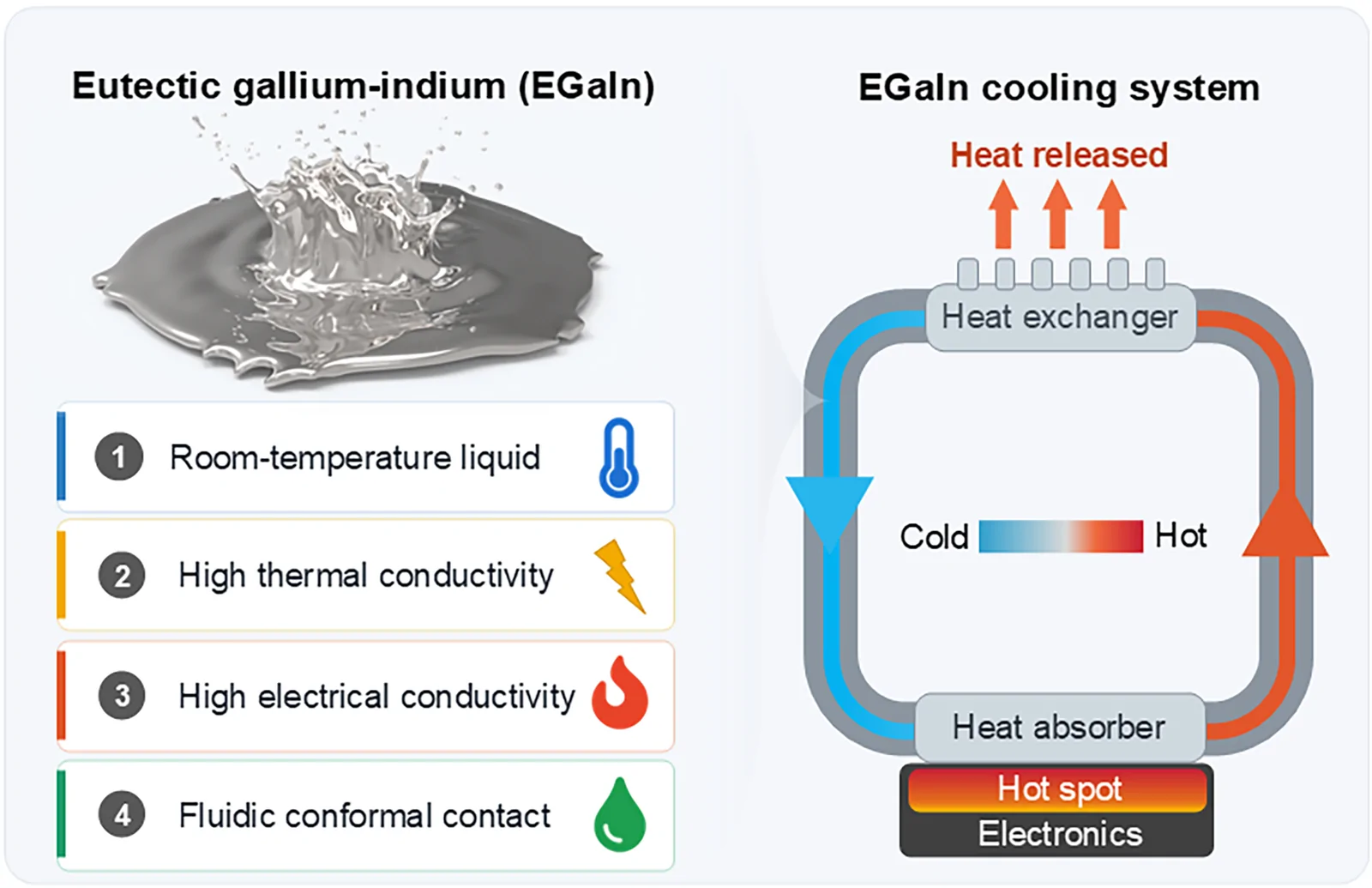
Concept of the eutectic gallium-indium (EGaIn) liquid metal cooling system (Left) EGaIn and its four key properties as a liquid metal cooling (right) schematic of the closed-loop cooling system. EGaIn absorbs heat from a hotspot on the electronics at the heat absorber. The heated liquid metal (red) circulates upward to the heat exchanger, where the heat is released to the surroundings. The cooled liquid metal (blue) then returns to the heat absorber to repeat the cycle. The color bar indicates the EGaIn temperature from cold to hot.

### Room-temperature fluidity and high thermal conductivity

One of the most distinctive features of EGaIn is the simultaneous presence of liquid-like deformability and metal-like thermal conductivity. EGaIn remains liquid near room temperature while exhibiting a thermal conductivity of 26 W/m·K, substantially exceeding that of conventional cooling fluids (Table 1).^45^ This unique combination of properties enables EGaIn to bridge the gap in the design space between solid thermal conductors and liquid coolants. Conventional solid materials often provide high thermal conductivity but exhibit limited conformability when interfacing with rough or irregular surfaces.^65^^,^^66^ As a result, microscopic voids remain at the interface and increase thermal resistance. For example, a dry solid interface exhibited a contact thermal resistance of 1.17 cm^2^ K/W at 50 psi, whereas the use of thermal grease reduced this value to 0.14 cm^2^ K/W under the same pressure.^31^ However, conventional greases typically suffer from relatively low thermal conductivity. In contrast, water and other common cooling fluids can readily conform to contacting surfaces but possess thermal conductivities of only about 0.6 W/m·K at 298 K.^64^^,^^67^ Therefore, EGaIn occupies a distinct material space by combining liquid-like conformity with metallic thermal transport.

EGaIn combines the advantages of both material classes. Its fluid nature allows it to penetrate microscopic surface irregularities and eliminate air-filled gaps, while its metallic thermal conductivity establishes efficient heat-transfer pathways across the interface.^29^ Because interfacial air layers represent a major source of thermal resistance, replacing them with EGaIn significantly improves heat transfer and reduces overall interfacial resistance.^5^ Accordingly, the combination of fluidity and high thermal conductivity provides the fundamental basis for EGaIn-mediated interfacial heat transfer. In practical interfaces, however, this liquid-mediated pathway is further influenced by the native oxide skin that forms spontaneously on EGaIn.

Within this liquid-mediated pathway, the native GaO_x_ skin, composed predominantly of Ga_2_O_3_, introduces a serial resistance associated with conduction through the oxide and thermal boundary resistance at the substrate-oxide and oxide-EGaIn boundaries.^48^^,^^51^^,^^64^ The thermal conductivity reported for amorphous Ga_2_O_3_ fragments is 0.1–0.3 W/m·K, substantially lower than the 26 W/m·K of bulk EGaIn.^42^^,^^45^ Using this reported range and a native oxide thickness of 0.7–3 nm gives an estimated area-normalized through-film resistance of 0.002–0.03 mm^2^ K/W per oxide-covered interface.^68^ This estimate represents conduction through an ideal continuous oxide film and excludes the two thermal boundary resistances and additional resistance associated with folded oxide, incomplete wetting, and residual voids. Thus, although the intrinsic resistance of intact nanometer-scale oxide is relatively small, changes in its thickness, morphology, and interfacial contact can increase its contribution to the overall thermal contact resistance (TCR).

The oxide skin governs the pressure-dependent conformity of EGaIn measurements report a critical surface stress of 0.5–0.6 N/m, depending on the deformation mode and test geometry.^45^^,^^48^ Below this threshold, the oxide stores stress elastically and resists spreading. Once the threshold is exceeded, yielding or local rupture allows the low-viscosity liquid core to redistribute, enter surface valleys, and displace residual gaps.^45^ Because this quantity is a surface stress rather than a bulk pressure, the corresponding pressure depends on the characteristic surface length scale. An EGaIn microchannel study reported PcL ≈1.1 N/m, corresponding to 110 kPa for a 10 μm feature.^45^^,^^48^ Although this value is not a universal TIM assembly pressure, it demonstrates that applied pressure can reduce TCR by decreasing the BLT, overcoming oxide-supported stress, and increasing the real EGaIn-solid contact area.

The resulting wetting response is strongly substrate dependent. Under an HCl-reducing atmosphere, the contact angle of EGaIn decreased from 105° to 30° on Cu but only from 117° to 100° on silicone.^69^ Reactive wetting and CuGa_2_ formation promoted spreading on Cu, whereas EGaIn retained poor wettability on silicone after oxide removal.^69^ As a result, improved wetting reduces the fraction of residual interfacial voids and the associated gap resistance, whereas incomplete wetting preserves voids that impede interfacial heat transfer. The resulting TCR is determined by the coupled effects of substrate chemistry, oxide state, surface roughness, BLT, and contact pressure under the specified interface conditions.^29^^,^^69^

### Comparison of EGaIn and water cooling for high heat flux cooling

The thermal management performance of EGaIn relative to water depends on the dominant thermal resistance and the scale of the cooling system. EGaIn is particularly advantageous in confined, interface-limited configurations, where interfacial conduction and localized heat spreading govern the temperature rise. By contrast, water generally remains advantageous for large-area convective heat removal in macroscopic systems because its much higher specific heat capacity provides greater sensible-heat transport capacity.^58^^,^^64^^,^^70^ Recent studies of EGaIn-based materials and interconnections show that EGaIn is effective in confined, interface-limited systems. An engineered tannic acid-mediated Cu-EGaIn composite achieved a thermal conductivity of 66 W/m·K,^40^ while a structured Cu-EGaIn-Cu interconnection exhibited a total area-normalized thermal resistance of 2.5–3.0 mm^2^ K/W.^69^ These engineered interfaces demonstrate that EGaIn can provide rapid heat spreading and low-resistance heat extraction near localized sources, but the reported values should not be interpreted as intrinsic properties of bulk EGaIn. When localized hotspots develop near an interface, minimizing the initial thermal resistance between the heat source and the cooling pathway becomes particularly important. Under these conditions, EGaIn rapidly distributes thermal energy away from the hotspot through conductive heat spreading, which reduces local temperature gradients and suppresses temperature accumulation.^69^ Therefore, EGaIn should be regarded as a context-specific complement to water cooling rather than a universal replacement. It is favored when interfacial conduction and hotspot spreading dominate, whereas water is generally favored when large-area convective transport and bulk heat capacity dominate.^18^^,^^70^

### Eutectic composition and operational stability

Although pure Ga exhibits favorable thermal properties, its melting point of 303 K creates operational challenges because phase transitions can occur at or near ambient conditions.^5^ Partial solidification may occur during device startup,^71^ storage,^72^ or operation under fluctuating environmental conditions,^73^ disrupting interfacial contact and reducing heat-transfer continuity.^74^ EGaIn overcomes this limitation through its eutectic composition, which lowers the melting temperature to 289 K (Table 1).^45^ A eutectic alloy corresponds to a specific compositional ratio at which the melting temperature reaches its minimum value, lower than that of any individual constituent. Although pure indium (In) melts at about 430 K, EGaIn undergoes a single, reproducible phase transition rather than melting over a broad temperature range. This low eutectic temperature provides a significant practical advantage because EGaIn remains in the liquid state throughout most storage and operating conditions encountered in electronic systems. EGaIn can maintain continuous thermal contact, preserve interfacial adaptability, and sustain stable heat-transfer pathways without requiring external heating or phase-control strategies.^5^^,^^22^

### Electrical conductivity for active control and electrical isolation

The high electrical conductivity of EGaIn introduces both opportunities and challenges for thermal management design. On the one hand, electrical conductivity provides a basis for electrically driven control of EGaIn. In the EGaIn system discussed in the section on forced convective heat transfer, an applied electrical potential induced reversible surface oxidation and reduction. This redox process modulated the interfacial tension and produced oscillatory droplet motion.^75^ This field-responsive behavior extends EGaIn beyond passive heat transfer and provides a basis for adaptive cooling. Electrical conductivity also provides the material basis for electromagnetic pumping, in which current passing through a conductive liquid interacts with an applied magnetic field to generate a Lorentz body force that drives bulk circulation. Xiang et al. demonstrated this pumping mechanism using Ga_61_In_25_Sn_13_Zn_1_ rather than EGaIn.^76^ Their results are treated only as related-alloy evidence for the transferable pumping mechanism and not as direct EGaIn performance. This finding provides a mechanistic basis for considering electromagnetic pumping as a potential active-flow-control strategy for EGaIn, as it demonstrates that Lorentz forces can drive bulk circulation in a conductive Ga-based liquid metal. However, differences in alloy composition may lead to differences in electrical conductivity, viscosity, and other properties governing electromagnetic forcing and fluid flow. Therefore, while the underlying pumping mechanism may be transferable to EGaIn, the quantitative pumping performance reported for Ga_61_In_25_Sn_13_Zn_1_ cannot be directly attributed to EGaIn. EGaIn testing is required before the reported electromagnetic-pump performance can be generalized to EGaIn. On the other hand, the same electrical conductivity creates significant reliability concerns when EGaIn is integrated into electronic devices. Direct contact between EGaIn and conductive circuitry may result in current leakage, short circuits, and catastrophic device failure.^36^ Accordingly, practical EGaIn-based thermal management systems must be designed to maintain close thermal coupling while simultaneously ensuring electrical isolation from active electronic components.^38^ To address these challenges, various engineering approaches, including insulating layers,^77^ encapsulation structures,^78^ protective coatings,^79^ and anti-leakage architectures,^80^ have been developed to improve electrical safety and operational reliability. The practical utility of EGaIn cannot be evaluated solely on the basis of its intrinsic material properties. Instead, successful implementation depends on how effectively its advantageous characteristics, including fluidity and electrical conductivity, are controlled within real device architectures.^36^^,^^38^ While fluidity improves interfacial conformity, it also introduces leakage risks, whereas electrical conductivity enables active thermal control while simultaneously creating electrical safety concerns. Therefore, the performance of EGaIn-based thermal management systems is fundamentally governed by both material properties and engineering design.

## Thermal management approaches

The thermal management performance of liquid metals is governed by their intrinsic thermal conductivity and by how they are used within a given system.^81^ Depending on the system architecture, liquid metals can be used as interfacial thermal conductors, convective coolants, circulating heat-transfer media, or thermally conductive components within composite materials.^55^^,^^82^ Their effectiveness is influenced by factors such as surface roughness,^29^ interfacial contact quality,^83^ coolant flow behavior,^18^ and structural integration.^36^ At solid-solid interfaces, liquid metals improve thermal transport by filling microscopic gaps and increasing the effective contact area.^79^ In microchannel and loop-based cooling systems, they remove heat through a combination of conduction and fluid motion.^18^^,^^70^ When incorporated into composites and phase-change materials (PCMs), liquid metals establish thermally conductive pathways, facilitate heat spreading, and contribute to temperature regulation.

### EGaIn as a thermal interface material

Interfacial thermal resistance originates largely from incomplete contact and air-filled gaps between rough surfaces. EGaIn addresses this limitation through its combination of high thermal conductivity, fluidity, and mechanical compliance, enabling it to infiltrate surface irregularities, replace insulating air gaps, and increase the effective contact area (Figure 2A).^29^^,^^79^ Thus, EGaIn primarily reduces gap resistance while forming additional solid-EGaIn-solid heat-transfer pathways around surface asperities. On laser-micropatterned Cu surfaces, EGaIn infiltrated dense, shallow microgrooves and promoted the removal of trapped air, reducing the area-specific thermal interface resistance from 1.5 ± 1.1 to 0.37 ± 0.19 mm^2^ K/W.^29^ However, low interfacial resistance is not guaranteed by high intrinsic thermal conductivity alone. Insufficient wetting can preserve residual voids and limit gap filling.^81^ Therefore, effective EGaIn thermal interfaces require coordinated control of material properties, surface structure, wetting, and interfacial penetration.

**Figure 2:**
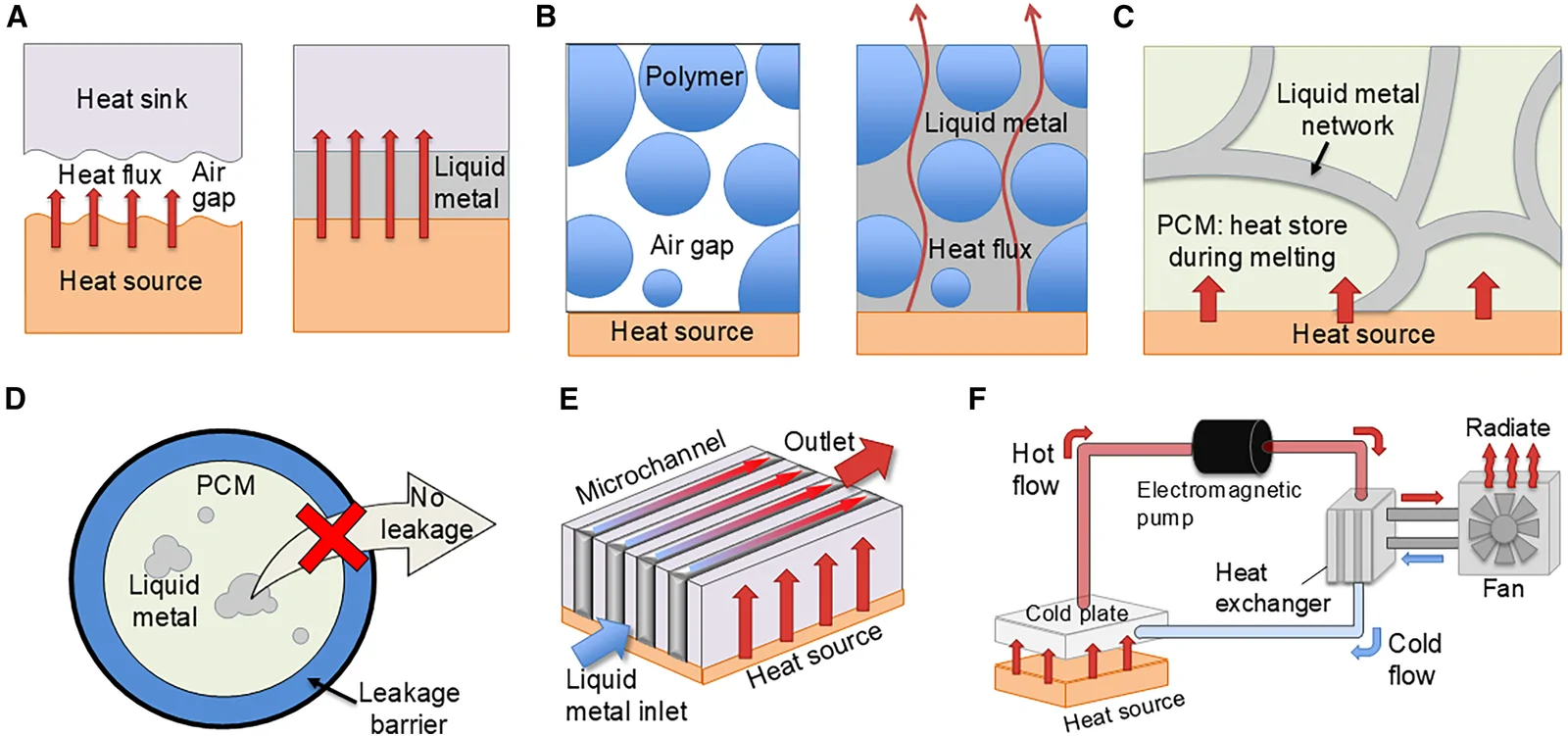
Liquid metal cooling strategies (A) (Left) interfacial thermal resistance reduction. Microscopic roughness between a heat source and heat sink traps insulating air gaps that impede heat flow. (Right) filling these gaps with liquid metal replaces the air pockets with thermally conductive pathways and increases the effective contact area. (B) Hybrid heat-spreading network in a composite. Insulating air gaps in the composite (left) are bridged by liquid metal to form continuous three-dimensional heat-conduction pathways (right). (C) Phase-change material (PCM)-assisted transient thermal buffering. Liquid metal networks enhance heat spreading, while the surrounding PCM absorbs latent heat during melting and delays temperature rise. (D) Leakage suppression and structural stabilization. A leakage barrier encapsulates the liquid metal-containing PCM to prevent leakage during phase change while preserving thermal performance. (E) Forced convective heat transfer in a microchannel heat sink, heat transferred from the heat source is carried away by liquid metal absorbing heat at the cold loop. (F) Loop-based heat transport, in which liquid metal circulated by an electromagnetic pump absorbs heat at a cold plate, releases it at a remote heat exchanger/fan, and returns to the source in a closed thermal circuit.

### EGaIn-based composites for thermal management

As an alternative to pure liquid metal, there have been efforts to create liquid metal-based composites composed of EGaIn containing fillers suspended within a soft polymer matrix. EGaIn containing fillers enhance heat transfer by forming continuous conductive pathways within the host matrix (Figure 2B).^82^ Although liquid metals possess intrinsically high thermal conductivity, efficient heat transport requires the establishment of interconnected networks that facilitate thermal conduction throughout the composite.^75^ The interfacial thermal resistance between isolated liquid metal inclusions and the polymer matrix can impede heat transfer from one inclusion to another. One strategy involves using a Cu particle in EGaIn (CuP-EGaIn) filler to bridge the gaps between neighboring thermoplastic elastomer (TPE) particles and reduce thermal resistance between adjacent conductive domains.^82^ In CuP-EGaIn/TPE composites, mechanical loading during heating causes the CuP-EGaIn filler to flow into interparticle gaps and merge into continuous conductive pathways. This process establishes three-dimensional (3D) heat-transfer networks throughout the polymer matrix.

### EGaIn-based PCM

PCMs provide thermal regulation by absorbing latent heat during melting and delaying temperature rise under transient thermal loads (Figure 2C).^8^^,^^84^ However, their generally low thermal conductivity limits heat spreading through the material. EGaIn evidence was reported by Xu et al., who incorporated Cu-coated graphite flakes into an EGaIn matrix and used hot pressing to form an interconnected liquid metal network within a phase-change composite.^85^ The resulting composite reached a thermal conductivity of 7.2 W/m·K at 50 vol % metallic filler, demonstrating that EGaIn can bridge adjacent conductive components and improve heat transfer through the phase-change matrix.^85^ A related battery study used a polyethylene glycol, Galinstan, and boron nitride (BN) composite and reported a reduction of more than 10 K in the maximum battery temperature.^8^ Because this system contains Galinstan rather than EGaIn, it is retained only as related-alloy evidence for a transferable filler-bridging architecture, and its quantitative battery performance is not treated as EGaIn evidence.

### Leakage suppression and structural stabilization

Although fluidity is central to the thermal advantages of liquid metals, it also introduces challenges related to leakage and structural instability.^35^^,^^79^ Consequently, effective containment strategies are essential for practical implementation. In electronic devices such as smartphones and wearable systems, liquid metal leakage can compromise thermal performance, contaminate adjacent components, and increase the risk of electrical failure.^36^ To address these issues, specialized anti-leakage architectures have been developed. One example employs In-bismuth-tin (In_51_Bi_32.5_Sn_16.5_)/In/In_51_Bi_32.5_Sn_16.5_ sandwich structure, in which In serves as a stabilizing layer while micropillar surface arrays improve wetting and mechanical retention.^36^ This design simultaneously suppresses oxidation and reduces liquid metal escape from the interface. Leakage is also a concern in PCM-based systems because the PCM itself becomes fluid during melting.^84^ Microencapsulation has therefore emerged as an effective strategy for improving structural stability (Figure 2D).^81^ By enclosing the PCM within protective shell materials, microencapsulation minimizes leakage, mitigates corrosion-related issues, and accommodates volume changes associated with phase transitions. These stabilization approaches are critical for maintaining long-term reliability while preserving the thermal advantages of liquid metal-containing systems.

### Forced-convection cooling

Forced-convection cooling uses externally driven fluid motion to transport heat away from high-power-density devices.^18^^,^^70^^,^^86^ In microchannel systems, heat is conducted from the device to the channel wall and then transferred to the moving coolant, while the high surface-area-to-volume ratio promotes wall-to-fluid heat exchange (Figure 2E).^87^ The high thermal conductivity of Ga-based liquid metals can enhance this heat exchange, although the overall performance also depends on heat capacity, viscosity, flow conditions, and channel geometry.^18^^,^^86^^,^^88^ EGaIn evidence for convection enhancement has instead been reported in an oscillating-droplet configuration rather than in a circulating EGaIn microchannel. Jeong et al. electrochemically actuated an EGaIn droplet positioned on a localized hotspot, generating vortices in the surrounding sodium hydroxide (NaOH) solution. The study identified this oscillation-induced flow as the primary heat-removal pathway, with conduction through EGaIn providing a secondary contribution. Through the combined effects of these heat-removal mechanisms, the hotspot temperature was reduced by 20% relative to the condition containing only NaOH, with the highest cooling efficiency observed at 3 Hz.^75^ The oscillation was generated by applying a ± 2 V square-wave potential in 1.0 M NaOH and required an actuation power of 0.04 W. Reversible surface oxidation and reduction modulated the interfacial tension, producing repeated spreading and recoil of the EGaIn droplet.^75^ Although the voltage waveform and frequency are electronically adjustable, the reported experiments employed prescribed frequencies under fixed heating conditions and did not demonstrate closed-loop adaptation to time-varying or spatially shifting thermal loads. Integrating real-time hotspot sensing with feedback control of the actuation waveform, frequency, or selected droplets within an array therefore represents an important next step toward adaptive cooling. Studies of T-Y and manifold microchannels using GaInSn are retained as related-alloy evidence that branched and manifold flow paths can improve coolant distribution under high heat flux.^6^^,^^87^ However, because viscosity, heat capacity, wetting, and material compatibility vary with alloy composition, their quantitative performance is not treated as EGaIn performance. Thus, EGaIn experiments are required before the quantitative benefits of these microchannel architectures can be generalized to EGaIn cooling.

### Loop-based heat transport

Loop-based cooling systems transport thermal energy from a localized heat source to a remote heat exchanger by continuously circulating a coolant through a closed thermal circuit (Figure 2F).^18^^,^^70^^,^^89^ For EGaIn, this architecture links three intrinsic properties to distinct thermal-management functions. Its eutectic melting point near 289 K supports liquid operation near room temperature, its thermal conductivity of 26 W/m·K promotes rapid heat uptake and redistribution within the coolant, and its electrical conductivity of 3.4 × 10^6^ S/m enables electrically driven flow control.^45^^,^^58^^,^^61^ These combined properties allow EGaIn to function as both a heat-transfer medium and an actively controllable coolant within compact circulation loops.

Once heat is absorbed near the source, continuous EGaIn circulation transports the thermal energy to a remote heat exchanger and returns the cooled liquid to the heated region. Because the specific heat capacity of EGaIn is 350 J/kg·K, compared with 4,180 J/kg·K for water at 298 K, the EGaIn mass flow rate becomes an important design variable governing the heat-transport capacity of the loop.^45^^,^^64^ Electromagnetic pumping provides a means of controlling this circulation without mechanically moving pump components. Current passing through the conductive liquid interacts with an orthogonal magnetic field to generate a Lorentz body force, J × B, that drives the coolant through the loop.^76^

Xiang et al. experimentally demonstrated this pumping architecture using G_a61_In_25_Sn_13_Zn_1_, producing a maximum pressure head of 30.9 kPa and a flow rate of 2.85 L/min at 400 A while supporting a heat flux of 293 W/cm^2^ near 343 K.^76^ Because the working fluid differed from EGaIn, these numerical values are treated as architecture-level evidence rather than EGaIn performance. Nevertheless, the electromagnetic pumping mechanism is applicable to EGaIn because its high electrical conductivity supports current transport and Lorentz-force generation. An EGaIn-specific electromagnetic loop can therefore integrate near-room-temperature fluidity, rapid conductive heat uptake, and electrically regulated circulation to transport localized heat loads to a remote heat exchanger.

## Applications

The applications discussed below arise directly from the EGaIn property-to-mechanism relationships established above. Its near-room-temperature fluidity and high thermal conductivity support conformal thermal interfaces, continuous heat-spreading networks, and rapid heat uptake from localized sources.^29^^,^^45^^,^^69^ Its electrical conductivity provides the basis for electrochemically driven droplet motion and electromagnetic circulation, while its native oxide regulates wetting, adhesion, and interfacial contact.^48^^,^^75^^,^^76^ Accordingly, EGaIn can function as a stationary interfacial medium in TIMs and composites, a field-responsive coolant for localized hotspot mitigation, and a circulating working fluid in battery, microchannel, and power-electronics cooling architectures. The following sections relate each application to the EGaIn properties and heat-transfer mechanisms that enable it.

### Active and passive battery thermal management

The rapid development of high-performance electronic devices and electrified transportation systems has intensified the demand for advanced battery thermal management technologies.^22^^,^^90^ Efficient thermal regulation is essential because excessive heat accumulation accelerates electrochemical degradation, reduces battery lifetime, and increases the risk of thermal runaway.^90^^,^^91^^,^^92^^,^^93^ Building on these intrinsic properties, EGaIn-based thermal management strategies have been explored for battery systems through both active and passive cooling architectures. Current battery-cooling designs using EGaIn can generally be categorized into two distinct configurations: active direct-circulation systems and passive composite-based cooling systems (Figures 2F and 3A).^94^^,^^95^^,^^96^^,^^97^

**Figure 3:**
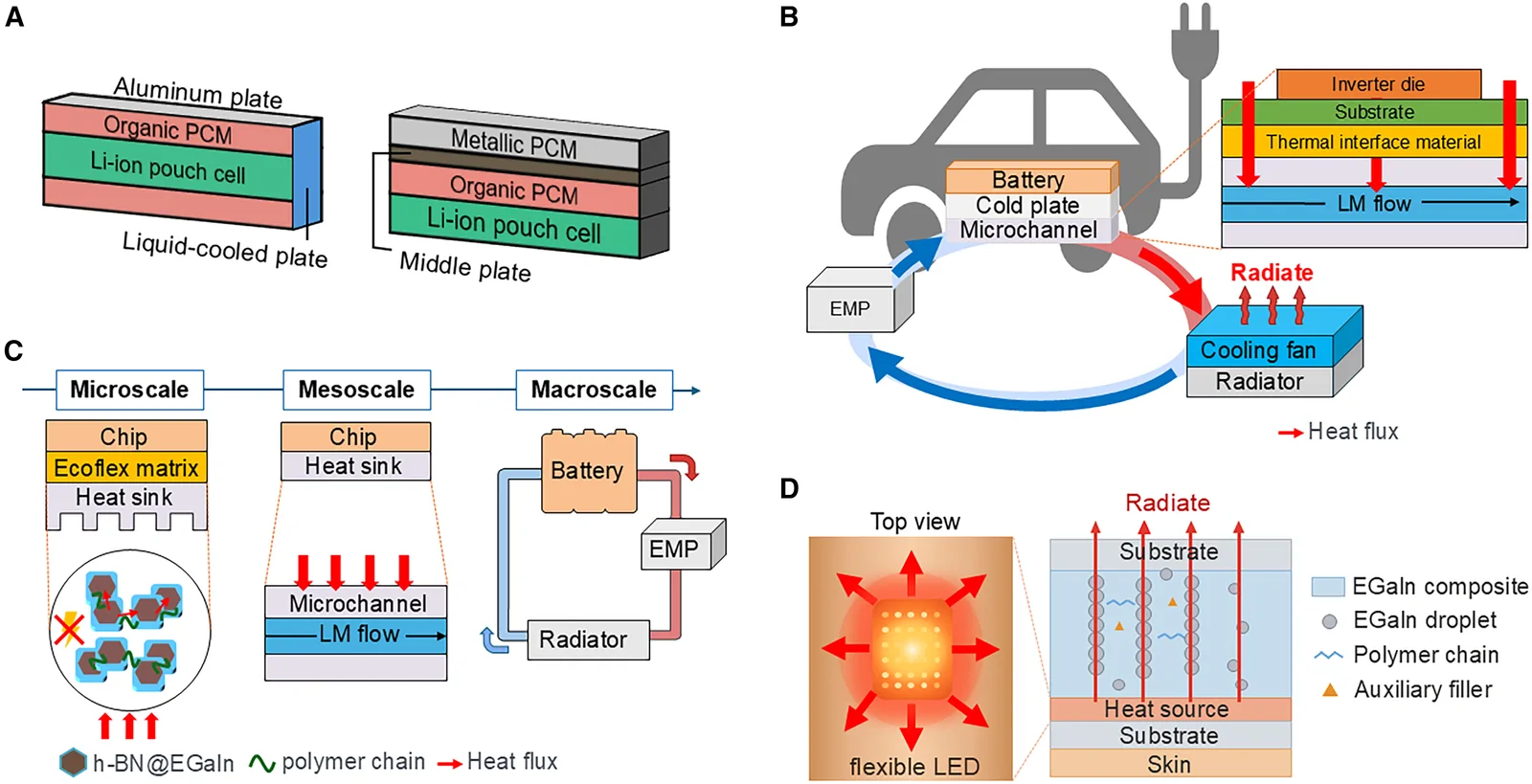
Applications of EGaIn-based thermal management in electronic and energy systems (A) Phase-change material (PCM)-based battery cooling with organic and metallic PCM layers for heat storage and reduced thermal resistance in a lithium (Li)-ion pouch cell. (B) Active eutectic gallium-indium (EGaIn) cooling of electric vehicle (EV) batteries and power electronics through EGaIn circulation in a cold plate, followed by heat rejection at a radiator. (C) Multiscale cooling concepts, including (microscale) chip-level h-BN@EGaIn hybrid filler embedded Ecoflex composite functioning as a thermal interface material (TIM), (mesoscale) microchannel cooling, and (macroscale) battery-to-radiator loop cooling. (D) Flexible electronics cooling with an EGaIn composite layer for heat spreading from a flexible light-emitting diode (LED) while maintaining flexibility.

In active direct-circulation systems, EGaIn functions as a flowing coolant that continuously transports thermal energy away from battery cells. These systems exploit exceptionally low Prandtl number of EGaIn (Pr ≈ 0.030),^98^ which is substantially lower than that of water (Pr ≈ 6.99).^99^ The Prandtl number describes the relative rates of momentum and thermal diffusion and is an important parameter governing heat-transfer behavior in fluid-based cooling systems. Because thermal diffusion in EGaIn occurs much more rapidly than momentum diffusion, heat can be distributed throughout the coolant more efficiently than in conventional liquids. EGaIn is circulated through microchannels positioned between adjacent battery cells using an external pumping system (Figure 2F). As heat generated within the cells is transferred to the flowing liquid metal, thermal transport within the coolant becomes increasingly dominated by conductive diffusion rather than solely by convective transport. This behavior promotes rapid temperature equalization across the battery module and reduces thermal gradients between individual cells. Yang et al. demonstrated that this mechanism effectively suppresses peak battery temperatures below thermal-runaway thresholds, even under discharge rates of 1 C –4 C.^9^ Despite these thermal advantages, active liquid metal cooling systems face several engineering challenges. The high surface tension of EGaIn (∼0.624 N/m) contributes to significant capillary resistance and complicates fluid transport within confined channels.^45^ Furthermore, EGaIn’s high density and electrical conductivity generate substantial hydraulic pressure losses during circulation.^100^ As a result, greater pumping power may be required to maintain adequate flow rates, increasing parasitic energy consumption. Greater pumping power may be required to maintain adequate flow rates, increasing parasitic energy consumption and reducing overall system efficiency. In addition, continuous pressurized operation can accelerate sealing fatigue, while leakage of electrically conductive liquid metal introduces a potential risk of short circuits and system failure. These challenges highlight an inherent trade-off. The same physical properties that make EGaIn thermally effective as a coolant also complicate its safe and efficient circulation.

Rather than transporting heat through fluid flow, these systems exploit the high intrinsic thermal conductivity of EGaIn to enhance heat spreading within a stationary thermal management material. Guo et al. incorporated EGaIn as microscopic droplets into a BN-filled phase-change composite, where the droplets formed thermally conductive percolation networks that enabled rapid lateral heat transport, while the BN fillers preserved electrical insulation and prevented short-circuit formation.^8^ While specific literature on EGaIn in this exact configuration is currently limited, the efficacy of this concept has been well demonstrated using other Ga-based alloys. For example, Guo et al. incorporated Galinstan as microscopic droplets into a BN-filled phase-change composite.^8^ The liquid metal formed thermally conductive percolation networks that facilitated rapid lateral heat transport, while the BN fillers maintained electrical insulation and prevented short-circuit formation. As a result, the composite achieved thermal conductivities as high as 8.8 W/m·K while reducing battery temperatures by more than 10 K during high-rate charging and discharging processes. Although these exact values were derived from a Galinstan-based system, the underlying structural concepts and heat-transfer mechanisms are equally applicable to EGaIn. While the absolute thermal metrics may vary slightly due to the material differences, identical conceptual cooling effects and performance enhancements can be expected from EGaIn.

Liu et al. adopted a hybrid phase-change architecture in which incorporates approximately 25% of a conventional organic PCM was replaced by Ga (Figure 3A).^101^ The incorporation of the metallic phase significantly reduced the overall thermal resistance of the composite while preserving latent-heat storage capability. Under ultra-fast discharge conditions, the optimized system maintained the maximum battery temperature at 309 K and achieved a temperature reduction of 2.4 K compared with the reference configuration. These active and passive strategies reveal a consistent trade-off rather than simply offering distinct advantages. Active circulation systems provide rapid heat removal and excellent temperature uniformity under high thermal loads, whereas passive composite systems offer improved safety, structural simplicity, and electrical insulation. Future battery thermal management technologies may increasingly integrate both approaches to balance cooling performance, energy efficiency, and operational reliability.

### Microchannel chip cooling

Microchannel cooling systems based on EGaIn and related Ga-based liquid metals have attracted significant attention for the thermal management of high-heat-flux electronic devices and advanced microprocessors.^6^^,^^67^^,^^102^ As device integration continues to increase, conventional air-cooling and water-cooling technologies face growing limitations in dissipating localized hotspots generated within compact chip architectures.^103^ Their cooling performance is often constrained by the thermal transport capability of the coolant and the ability of the cooling structure to remove heat from confined regions.^104^ Ga-based liquid metals have emerged as promising coolants for high-heat-flux microchannel systems, particularly where conventional coolants struggle to suppress localized hotspots.^70^ EGaIn remains liquid near room temperature and can be integrated into microscale cooling architectures without requiring elevated operating temperatures, making it particularly attractive for next-generation electronic cooling systems.^45^ Although previous studies investigated related Ga-based alloys rather than EGaIn, the resulting microchannel designs offer valuable guidance for EGaIn-based systems because channel geometry, flow distribution, and hydraulic resistance are key determinants of cooling performance.^6^^,^^67^^,^^105^^,^^106^

Direct experimental support for EGaIn circulation was provided by Ye et al., who used EGaIn throughout their experiments with a closed microchannel cooling loop incorporating a split electromagnetic centrifugal pump driven by rotating permanent magnets.^107^ The system demonstrated controllable EGaIn circulation and cooling at heat fluxes up to 3.5 W/cm^2^, providing experimental support for the feasibility of EGaIn-based channel cooling. However, in conventional straight microchannels, the coolant follows a single extended flow path, causing a progressive temperature rise along the channel length.^67^^,^^108^ This limitation is particularly relevant to Ga-based liquid metals because their volumetric heat capacity is lower than that of water, resulting in a greater coolant temperature rise at a matched volumetric flow rate. To overcome this challenge, Zhang et al. numerically investigated a manifold microchannel heat sink using a Ga_68_In_20_Sn_12_ coolant rather than EGaIn.^6^ The manifold architecture divided the coolant flow into multiple shorter channels, reducing the flow-path length and improving thermal uniformity.^6^ At a heat flux of 1,000 W/cm^2^, the simulated maximum chip temperature remained below 352 K, whereas the conventional straight-channel model exceeded 440 K under comparable conditions. The heat-transfer coefficient reached 3.5 × 10^5^ W/m^2^·K in the baseline comparison and approached 1.0 × 10^6^ W/m^2^·K in the parametric study. Under the specified condition of equal heat-dissipation capacity, the water-cooled manifold required 5.2 times the pressure drop and 2.1 times the pumping power of the GaInSn design.^6^ Based on these design concepts, EGaIn microchannel systems can integrate the channel heat sink with mechanical or electromagnetic circulation and an external heat exchanger, with overall performance governed by flow rate, coolant properties, wetting, and channel geometry (Figure 2E).

Alternative microchannel architectures have also been explored to improve thermal-hydraulic performance. Using GaInSn coolant rather than EGaIn, Liu et al. investigated a T-Y-type microchannel heat sink incorporating branched T-shaped and Y-shaped flow passages.^87^ The branching structure promoted more uniform coolant distribution and dispersed high-temperature regions. The equivalent heat-transfer coefficient of reached 4.7 × 10^4^ W/m^2^·K at a pressure drop of 2.21 × 10^4^ Pa. Separately, a surface-temperature difference of 27 K was reported at a heat flux of 1.0 × 10^6^ W/m^2^.^87^ These results illustrate how channel geometry can influence heat-transfer efficiency and temperature uniformity and provide design guidance for prospective EGaIn systems.

Additional efforts have focused on optimizing microchannel structures to balance thermal performance and hydraulic losses. In a numerical study using Galinstan rather than EGaIn, Chen et al. evaluated top-slotted, bottom-extension, and composite microchannel configurations designed to improve coolant transport while minimizing flow resistance.^67^ Among the investigated designs, the composite microchannel exhibited the lowest hydraulic resistance and required less pumping power while maintaining heat-transfer performance comparable to that of the alternative configurations. These findings emphasize the importance of structural optimization and provide useful design guidance for future EGaIn systems. Despite the promising thermal performance predicted or observed for Ga-based liquid metal channel systems, several challenges continue to hinder their practical implementation.^82^^,^^105^ The high implementation pressure loss and pumping-power requirements are governed jointly by coolant density and viscosity, flow rate, channel geometry, and operating conditions.^51^ In addition, leakage prevention, long-term sealing reliability, electrical insulation, and material compatibility remain critical engineering concerns. Therefore, future development of EGaIn-based microchannel cooling technologies will require not only improvements in thermal performance but also advances in system reliability, containment strategies, and electrically safe device integration.

### Power electronics and EV inverter cooling

Power electronic systems, including IGBT modules, SiC MOSFETs, traction inverters, and electric vehicle (EV) power-conversion units, generate substantial heat loads that require effective thermal management to maintain performance and reliability.^109^^,^^110^^,^^111^ EGaIn-based cooling is attractive for these applications because its high thermal conductivity can support rapid heat extraction and transport within compact cooling architectures.^82^^,^^112^ Closed-loop configurations can couple a liquid metal-cooled cold plate to an external heat exchanger or radiator for continuous thermal regulation (Figure 3B).^110^

In 2021, Ma et al. investigated cooling technologies for high-power electronic systems, including EV inverters, wind-power converters, and variable-frequency refrigeration equipment.^111^ The authors demonstrated that liquid-cooling architectures can effectively dissipate heat generated within power modules while simultaneously improving temperature uniformity throughout the system. Enhanced cooling performance was attributed to the large heat-transfer area and improved thermal transport characteristics of the cooling structure, which reduced temperature gradients and facilitated more uniform heat removal. Although EGaIn was not directly employed in the reported system, the findings suggest that thermally conductive liquid metals could further improve cooling effectiveness in similar applications.

Reliable coolant circulation is also critical for power-electronics applications.^111^^,^^113^ Xiang et al. experimentally demonstrated a direct-current electromagnetic pump for Ga_61_In_25_Sn_13_Zn_1_, providing related-alloy evidence for mechanically passive circulation of conductive liquid metals.^76^ As discussed above, electromagnetic pumping exploits liquid metal electrical conductivity to generate Lorentz-force-driven flow without mechanically moving pump components, potentially reducing vibration, wear, and maintenance requirements.^113^ Although the reported quantitative performance cannot be directly attributed to EGaIn, its high electrical conductivity provides the basis for applying the same pumping principle to EGaIn-based cooling loops.

Despite the considerable promise of liquid metal cooling for power-electronic applications, several engineering challenges remain unresolved.^82^ Electrical insulation is particularly important because accidental leakage of conductive liquid metal can result in short circuits and catastrophic device failure.^40^ Additionally, corrosion, material compatibility, sealing reliability, and long-term operational stability must be carefully addressed before large-scale implementation becomes feasible.^112^^,^^113^ Furthermore, prolonged exposure to elevated temperatures and repeated thermal cycling may influence both coolant behavior and structural integrity, necessitating comprehensive reliability evaluation under realistic operating conditions.^114^ Existing studies indicate that liquid metal cooling systems have strong potential for thermal management of next-generation power electronics and EV inverter platforms.^109^^,^^115^ Their ability to rapidly remove heat, maintain temperature uniformity, and integrate with electromagnetic circulation technologies offers significant advantages over conventional cooling approaches. Nevertheless, continued advances in insulation strategies, corrosion mitigation, leakage prevention, and long-term reliability assessment will be essential for translating laboratory demonstrations into commercially deployable systems.

### Multiscale thermal management and packaging

Beyond its application as a circulating coolant, EGaIn has attracted considerable interest as a TIM and packaging component for highly integrated electronic systems.^29^^,^^116^^,^^117^^,^^118^^,^^119^^,^^120^^,^^121^^,^^122^^,^^123^ At the packaging level, minimizing interfacial thermal resistance must be balanced with electrical and mechanical reliability because the high electrical conductivity and fluidity of EGaIn can create leakage and short-circuit risks.^81^^,^^118^^,^^122^^,^^123^^,^^124^ Composite designs have therefore been developed to retain the thermal advantages of EGaIn while improving electrical insulation and structural stability.^118^^,^^119^^,^^120^^,^^121^^,^^122^^,^^123^ Fu et al. developed an h-BN@EGaIn/Ecoflex composite that functions as both a TIM and encapsulation layer (Figure 3C).^125^ In this structure, EGaIn was anchored to electrically insulating h-BN nanosheets through the adhesive Ga_2_O_3_ surface layer and acted as a thermal bridge between adjacent fillers, converting point-type contacts into more effective surface-type contacts. This architecture enhanced interfacial thermal transport while restricting EGaIn mobility and suppressing continuous electrically conductive percolation pathways. The composite achieved a thermal conductivity of 0.904 W/m·K compared with 0.163 W/m·K for pristine Ecoflex, while maintaining electrical insulation and limiting liquid metal migration under mechanical deformation.^125^ This approach demonstrates how controlled EGaIn immobilization within an insulating, mechanically compliant matrix can simultaneously enhance thermal transport and address leakage, flexibility, and electrical-safety requirements in electronic packaging.

At an intermediate length scale, liquid metal thermal management can be integrated into chip-adjacent microchannel structures for active hotspot cooling (Figure 3C). In 2024, Zhang et al. demonstrated a manifold architecture that divided the coolant flow into shorter channels, improving thermal uniformity while reducing hydraulic resistance.^6^ The simulated system dissipated heat fluxes approaching 1,000 W/cm^2^ while maintaining the maximum chip temperature below 352 K, demonstrating the potential of manifold-based liquid metal cooling for high-heat-flux electronics. At larger system scales, liquid metal cooling can extend beyond localized chip cooling to integrated heat-transport networks. Yang et al. demonstrated a closed-loop cooling system for EV battery applications that coupled localized heat removal with long-range thermal transport (Figure 3C).^9^ Mathematical and numerical analyses showed that liquid metal cooling reduced the intercell temperature difference to 2.3 K compared with 3.1 K for water cooling. At the same flow rate, the liquid metal system required approximately 40% of the pumping power of water cooling, while achieving the same target temperature at a 5-fold lower flow rate reduced pumping power by a factor of 25.^9^ These studies demonstrate the multiscale potential of liquid metal thermal management, ranging from microscale thermal interfaces and mesoscale hotspot cooling to macroscale heat-transport networks. Practical implementation across these scales requires thermal performance to be balanced with electrical safety, structural stability, and long-term reliability.

### Thermal management of soft electronics

The rapid expansion of wearable electronics, electronic skins, stretchable sensors, and soft robotic systems has created a growing demand for thermal management technologies that combine efficient heat dissipation with exceptional mechanical compliance.^15^^,^^126^^,^^127^^,^^128^^,^^129^ Unlike conventional rigid electronics, soft electronic devices are continuously subjected to bending, stretching, twisting, and compression during operation. Thermal management materials must therefore maintain efficient heat-transfer pathways while accommodating repeated mechanical deformation without degradation of thermal performance.^126^ Ga-based liquid metals have emerged as promising candidates because their room-temperature fluidity and deformability enable intimate contact with deformable structures while maintaining continuous thermal transport pathways.^125^^,^^130^ Depending on the cooling strategy, EGaIn can function either as a circulating coolant for active heat removal or as an embedded thermally conductive component within flexible composite materials.

Active liquid metal cooling provides an effective strategy for removing heat from localized high-power sources. Deng et al. investigated a Ga_80_In_20_-based closed-loop cooling system for thermal management of high-power LEDs.^70^ The system consisted of a Cu cold plate, circulation pump, and air-cooled radiator through which liquid metal continuously transported thermal energy away from the heat source. Heat generated by a 100 W LED module was absorbed by the circulating liquid metal and released through the external heat exchanger. Compared with water cooling under identical flow conditions (10.8 mL/s), the liquid metal system reduced the substrate temperature rise from 22 to 13 K while increasing the convective heat-transfer coefficient from 3.7 × 10^3^ to 9.3 × 10^3^ W/m^2^·K. The authors further demonstrated that the enhanced cooling performance primarily originated from the substantially higher convective heat-transfer coefficient of the liquid metal, which significantly reduced convective thermal resistance within the cooling loop. These results established the feasibility of liquid metal circulation for high-heat-flux thermal management and provided an important foundation for subsequent liquid metal cooling technologies.

While actively circulated liquid metals provide highly efficient cooling for localized heat sources, wearable and deformable electronic systems generally require passive thermal management materials that eliminate the complexity associated with pumps, tubing, and external heat exchangers (Figure 3D). Recent research has increasingly focused on incorporating EGaIn into stretchable composite materials capable of simultaneously providing high thermal conductivity, mechanical compliance, and long-term durability. Bartlett et al. showed that EGaIn microdroplets suspended in silicone could function as a heat spreader, with thermal conductivity approaching 10 W/m·K when the EGaIn-silicone composite is stretched to 400% strain.^38^ Because of its soft and elastic properties, the composite could be placed on human skin and used as a substrate for high-power electronics without trapping heat or causing discomfort. Along similar lines, Chen et al. developed an elastomeric composite containing dispersed EGaIn droplets that dynamically reorganized under applied strain to preserve continuous thermal-conduction pathways during mechanical deformation.^130^ The composite exhibited a thermal conductivity of 1.78 W/m·K while maintaining an elongation at break approaching 614%, demonstrating that efficient thermal transport can be maintained even under extreme stretching.

Further improvements in thermal performance have been achieved through hierarchical conductive architectures. Luo et al. fabricated a 3D graphene-coated EGaInSn architecture integrated within an aramid nanofiber (ANF) scaffold for flexible thermal management.^131^ Graphene nanosheets formed thermally conductive shells around EGaInSn nanodroplets, while the liquid metal bridged adjacent graphene domains to establish continuous thermal-conduction pathways throughout the composite. The resulting architecture exhibited an in-plane thermal conductivity of 5.67 W/m·K together with an interfacial thermal resistance as low as 3.78 × 10^−8^ m^2^ K/W, outperforming comparable liquid metal/ANF and graphene/ANF composites. Incorporation of EGaInSn also reduced the Young’s modulus by 24.5%, enabling efficient heat spreading without sacrificing mechanical flexibility. These results demonstrate that hierarchical liquid metal architectures improve thermal conductivity, reduce interfacial thermal resistance, and preserve mechanical compliance.

Liquid metal-based thermal management for soft electronics has evolved along two complementary directions. Active circulation systems provide rapid heat removal for localized high-power devices, whereas passive liquid metal composites and thermal interface materials provide mechanically compliant heat spreading for wearable and deformable electronics. Future developments are expected to emphasize electrically insulating composite architectures, leakage-resistant encapsulation, long-term mechanical durability under repeated deformation, and scalable manufacturing approaches that enable reliable integration of liquid metal thermal management technologies into next-generation soft electronic systems.

## Challenges and reliability of EGaIn thermal management

Although EGaIn offers unique advantages for thermal management by combining metallic thermal transport with liquid-like adaptability, several challenges limit its widespread implementation.^53^^,^^55^ Successful deployment requires stable thermal performance under realistic environmental, mechanical, and electrical conditions, with oxidation and containment representing important interfacial and mechanical concerns.^125^^,^^130^ Native Ga_2_O_3_ should be considered a functional but evolving interfacial state rather than solely a material limitation. A controlled oxide layer can support shape retention and substrate adhesion, whereas changes in its thickness, integrity, or chemistry modify EGaIn rheology, wetting, and contact with heat-transfer surfaces.^48^^,^^81^^,^^132^^,^^133^^,^^134^^,^^135^ Repeated oxide formation, rupture, and reformation can alter the effective contact area and residual voids, resulting in variability in TCR and actuation behavior.^38^^,^^81^^,^^125^^,^^132^^,^^134^^,^^135^ The liquid nature of EGaIn also creates risks of leakage, displacement, and pump-out during mechanical loading, vibration, deformation, and thermal cycling.^36^^,^^136^^,^^137^ Repeated deformation may induce fatigue or delamination of encapsulating interfaces, allowing gradual EGaIn migration before visible leakage occurs.^136^^,^^138^ Although encapsulation layers, elastomeric barriers, composite formulations, and structural confinement can improve retention,^125^^,^^131^^,^^136^ additional interfaces or insulating phases may compromise thermal performance.^139^ Thus, maintaining a reproducible oxide state and balancing containment reliability with heat-transfer efficiency are important requirements for stable operation.

Material compatibility represents another critical limitation because Ga-induced degradation strongly depends on the contacted material and its microstructure. Al is particularly susceptible to Ga penetration along grain boundaries and liquid metal embrittlement,^140^ whereas EGaIn exposure to Cu can cause reactive dissolution, interdiffusion, and formation of Cu-Ga reaction layers.^138^^,^^141^ Liquid metal embrittlement has also been reported for Cu-Zn brass under plastic deformation, although this behavior should not be generalized to pure Cu because susceptibility depends on composition, microstructure, wetting, and stress.^142^ Ga-induced degradation can additionally occur in Sn-based solder alloys.^143^ These observations emphasize the need for material-specific compatibility testing and protective coatings or diffusion barriers where direct EGaIn contact cannot be avoided.^141^

For actively circulated EGaIn, thermal performance must be considered together with hydraulic efficiency. Its relatively high density and viscosity can increase pressure losses and pumping requirements, while its moderate specific heat capacity can contribute to temperature rise along the flow path if circulation is not optimized.^6^^,^^45^^,^^58^^,^^67^^,^^144^^,^^145^ Material cost and Ga supply constraints may also become relevant for large-scale deployment.^90^^,^^146^ Furthermore, EGaIn should not be assumed to remain liquid below its equilibrium melting point of 289 K. Although supercooling can delay solidification, freezing depends on thermal history, confinement, oxide state, and nucleation conditions.^57^^,^^58^ Systems that may cross this temperature therefore require appropriate freeze-thaw qualification and accommodation of solidification-induced volume and structural changes.

Long-term reliability data under realistic operating conditions remain limited. Compared with studies reporting thermal conductivity and TCR, fewer investigations have systematically examined prolonged thermal cycling, high-heat-flux operation, repeated mechanical loading, or environmental exposure.^138^^,^^147^ Reliability assessment should therefore track application-relevant changes in thermal resistance, leakage, electrical isolation, interfacial integrity, and, for circulating systems, flow rate and pressure drop.^138^^,^^147^^,^^148^ Thermal cycling, freeze-thaw exposure, humidity, vibration, and deformation testing should be selected according to the intended architecture rather than treated as a universal EGaIn qualification protocol. Establishing application-specific reliability criteria, together with improved oxidation control, containment, material compatibility, and flow design, will be essential for translating the thermal advantages of EGaIn into robust thermal management technologies.

## Conclusion and future perspectives

EGaIn has emerged as a promising material for thermal management that combines high thermal conductivity with liquid-like deformability, enabling efficient heat transfer across interfaces and complex device architectures. EGaIn supports multiple cooling mechanisms, including interfacial thermal-resistance reduction, conductive heat spreading, convective cooling, loop-based heat transport, and thermally conductive composite formation. These capabilities make EGaIn attractive for applications ranging from battery thermal management and high-power electronics to chip packaging and flexible electronic systems.

Despite its considerable potential, several challenges continue to limit practical implementation. Oxidation, leakage, corrosion, electrical-safety concerns, and long-term mechanical reliability remain critical issues that must be addressed before large-scale deployment becomes feasible. Although various encapsulation strategies, composite designs, and protective structures have been proposed, their long-term performance under realistic operating conditions has not yet been fully established. Additionally, the lack of systematic reliability data obtained under prolonged thermal cycling, mechanical loading, and high-heat-flux operation continues to hinder the assessment of long-term device stability and commercial viability.

Future research should focus on maximizing thermal performance while improving material stability and reliability, especially under thermal cycling and cyclic mechanical loading. Key priorities include leakage-resistant encapsulation, corrosion-resistant interfaces, electrically insulating liquid metal composites, and standardized reliability-testing protocols. Furthermore, scalable manufacturing methods and system-level integration strategies will be essential for practical adoption. As thermal demands continue to increase in advanced semiconductor packaging, AI hardware, electric vehicles, and wearable electronics, adaptive thermal management technologies will become increasingly important. Beyond thermal management, the conductive and deformable nature of EGaIn may also support its integration with emerging energy-harvesting technologies that convert mechanical or thermal stimuli into electrical signals.^149^ With continued advances in materials design and reliability engineering, EGaIn-based systems have strong potential to contribute to next-generation thermal management solutions.

## Acknowledgments

This work was supported by the Academic Promotion System Tech University of Korea.

## Author contributions

Conceptualization, writing and editing, J.Y.J., H.N., Y.-H.J., S.H.Y., M.L., C.M., and H.K.; supervision, H.K.; all authors reviewed and approved the final manuscript.

## Declaration of interests

The authors declare no competing interests.
